# Great Apes and Biodiversity Offset Projects in Africa: The Case for National Offset Strategies

**DOI:** 10.1371/journal.pone.0111671

**Published:** 2014-11-05

**Authors:** Rebecca Kormos, Cyril F. Kormos, Tatyana Humle, Annette Lanjouw, Helga Rainer, Ray Victurine, Russell A. Mittermeier, Mamadou S. Diallo, Anthony B. Rylands, Elizabeth A. Williamson

**Affiliations:** 1 Department of Integrative Biology, University of California, Berkeley, California, United States of America; 2 The WILD Foundation, Berkeley, California, United States of America; 3 Durrell Institute of Conservation and Ecology, School of Anthropology and Conservation, University of Kent, Canterbury, United Kingdom; 4 Strategic Initiatives and Great Apes Program, The Arcus Foundation, Cambridge, United Kingdom; 5 Conservation Program, The Arcus Foundation, Cambridge, United Kingdom; 6 Business Conservation Initiative and Conservation Finance, Wildlife Conservation Society, Bronx, New York, United States of America; 7 Conservation International, Arlington, Virginia, United States of America; 8 Guinée Écologie, Conakry, Republic of Guinea; 9 Conservation International, Arlington, Virginia, United States of America; 10 Scottish Primate Research Group, School of Natural Sciences, University of Stirling, Scotland, United Kingdom; Instituto de Pesquisas Ecológicas, Brazil

## Abstract

The development and private sectors are increasingly considering “biodiversity offsets” as a strategy to compensate for their negative impacts on biodiversity, including impacts on great apes and their habitats in Africa. In the absence of national offset policies in sub-Saharan Africa, offset design and implementation are guided by company internal standards, lending bank standards or international best practice principles. We examine four projects in Africa that are seeking to compensate for their negative impacts on great ape populations. Our assessment of these projects reveals that not all apply or implement best practices, and that there is little standardization in the methods used to measure losses and gains in species numbers. Even if they were to follow currently accepted best-practice principles, we find that these actions may still fail to contribute to conservation objectives over the long term. We advocate for an alternative approach in which biodiversity offset and compensation projects are designed and implemented as part of a National Offset Strategy that (1) takes into account the cumulative impacts of development in individual countries, (2) identifies priority offset sites, (3) promotes aggregated offsets, and (4) integrates biodiversity offset and compensation projects with national biodiversity conservation objectives. We also propose supplementary principles necessary for biodiversity offsets to contribute to great ape conservation in Africa. Caution should still be exercised, however, with regard to offsets until further field-based evidence of their effectiveness is available.

## Introduction

Great apes–gorillas, chimpanzees, and bonobos–are distributed across 21 countries on the African continent [1]. Their conservation is important in several respects. Their geographic ranges are strongly associated with the tropical forests that harbor some of the richest biodiversity in the world and overlap extensively with those of many endemic species [2]. Great apes have large home ranges [3], and thus protection of their habitat will also bring many other species under protection. Great apes are keystone species, playing important roles in maintaining the health and diversity of their ecosystems through their seed dispersal [4]–[6]. In addition, they act as physical ecosystem engineers [7], [8] shaping the forest structure by trampling, bending and breaking vegetation as they travel, forage and build nests [9], [10]. Apes and ape habitat are important for people; protecting ape habitats protects important water catchment areas. In Rwanda, for example, the Volcanoes National Park provides much of that country’s water [11], and the Fouta Djallon in Guinea is the source of a number of West Africa’s major rivers, including the Niger. Tourism with great apes also provides significant income to local communities through revenue sharing and local businesses, thus providing livelihood opportunities for local people [12].

All great ape taxa are listed as either Endangered (EN) or Critically Endangered (CR) by the International Union for Conservation of Nature (IUCN) [13]. Threats to great apes include habitat loss, hunting and disease [13]. These threats are exacerbated by large-scale development activities such as hydroelectric projects, roads, and extractive industries [14], all of which result in the destruction of large areas of ape habitat and provide access to remote areas, facilitating bushmeat hunting [15]. Industrial development projects result in large influxes of people, exposing great apes to human diseases that can be fatal to them [16]. Human presence and activity can cause apes to leave their habitual ranges, which can result in competition, conflict and stress, with long-term consequences for the health and reproduction of the population [17], [18]. Mortalities are likely to occur if chimpanzees are forced into an area that is already occupied by conspecifics because chimpanzees are highly territorial and often attack intruders [19]. The effects of development projects are intensified for great apes because of their reproductive biology and slow maturation [14]. Even low levels of disturbance to ape populations can result in declines that require decades for their subsequent recovery [20].

Industrial development is proliferating throughout Africa [21], [22]. The interface between development projects and great ape conservation will, therefore, intensify in coming decades. Most countries in Africa where great apes occur rank high on the United Nations poverty index and are undergoing intensive infrastructure development [23]. A mining boom is occurring [24], [25], which will result in the expansion of transportation infrastructure [25]. More than 50% of the range of chimpanzees and gorillas in Western Equatorial Africa has been allocated to logging concessions [26]. The extensive overlap between the distribution of commodities, biodiverse areas and great ape ranges means that companies will increasingly need to mitigate the negative impacts of their projects on great ape populations [14], [27].

Options for mitigating impacts on great apes are limited. Relocation is risky [28] and can lead to mortalities [29], [30], and restoring habitat is not feasible on a time-scale meaningful to great apes [31]. Unless great ape habitat is avoided entirely, in most cases mitigation is unlikely to prevent great ape losses and most projects will result in some population decline.

“Biodiversity offsets” are increasingly used worldwide to compensate for the negative impacts of development and private sector projects on biodiversity [31]–[40]. The Business and Biodiversity Offset Program (BBOP), a broad consortium including civil society and private sector organizations, financial institutions, governments, and intergovernmental organizations, defines biodiversity offsets as “measurable conservation outcomes resulting from actions designed to compensate for significant residual adverse biodiversity impacts arising from project development after appropriate prevention and mitigation measures have been taken” [36]. According to BBOP, the goal of biodiversity offsets is “to achieve no net loss and preferably a net gain of biodiversity on the ground with respect to species composition, habitat structure, ecosystem function and people’s use and cultural values associated with biodiversity” [36]. Offsets are distinct from the broader category of biodiversity compensation projects, which mitigate impacts but do not follow a mitigation hierarchy or comply with other offset requirements [36].

Seventeen countries worldwide have national policies requiring biodiversity offsets, and more than 29 countries have national policies that suggest or enable the use of offsets [41]. No countries in the range of great apes in West and Central Africa, however, currently have policies guiding or requiring offsets [41]. Biodiversity offsets are therefore guided by private sector internal standards or those of lenders, rather than by government policy [42]–[45]. Several international organizations such as BBOP, the International Council on Mining and Metals (ICMM) and IUCN have proposed best practices for biodiversity offsets [36], [46]. BBOP best practices include a list of 10 guiding “principles” for the design and implementation of biodiversity offsets. These principles are reflected in many national offset policies around the world [34], in the scientific literature [31]–[35], [37]–[39], [47], in private sector internal guidelines [42], and in other international best practices [46], and are reflected in the lending standards and principles of many of the largest international banks [45], [46].

Despite this new emphasis on biodiversity offsets, there is little empirical evidence from the field to demonstrate that they are achieving conservation objectives over the long term. This is a result of the lack of standardized evaluation criteria, limited monitoring of projects, under-reporting of projects that are not working [31], and lack of access to information on projects due to confidentiality of reports. In addition, many offsets projects are still in the design or early implementation phase and do not yet have results to report. Biodiversity offset policies are still in their infancy [38], and more reviews of field projects are urgently needed before encouraging their wider use as a conservation tool [37], [38].

To assess how well field projects adhere to international best practice principles, we examine four projects in Africa where private sector or development projects are impacting great apes and their habitat and are seeking to use offsets and compensation projects to counterbalance these impacts. We measure them against the six BBOP principles related to biological criteria: (1) limits to what can be offset, (2) adherence to the mitigation hierarchy, (3) additional conservation outcomes, (4) landscape context, (5) no net loss, and (6) long-term outcomes. We also assess whether full compliance to these principles would be sufficient to generate conservation benefits for great apes in light of their EN and CR status, their shrinking habitat, and their vulnerability to disturbance. Although this paper focuses on great apes, we believe the results of this study will also apply to many other EN and CR taxa.

## Case Studies

**Figure 1:**
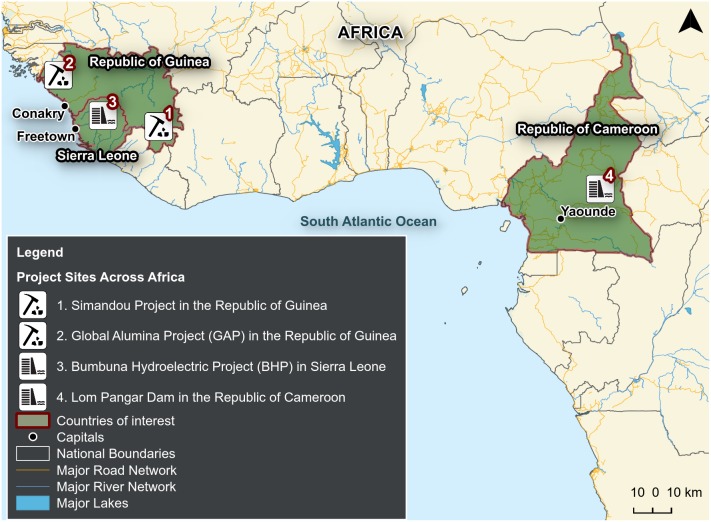
Sites in Africa where private sector or development projects are seeking to use offsets and compensation projects to counterbalance residual negative impacts to great apes and their habitat. Sites include (1) the Simandou Project in the Republic of Guinea, (2) the Global Alumina Project (GAP) in the Republic of Guinea, (3) the Bumbuna Hydroelectric Project (BHP) in Sierra Leone, and (4) the Lom Pangar Dam in the Republic of Cameroon.

We examine four projects in Africa that are investigating either biodiversity offsets or compensation for residual impacts to great apes and their habitat, or have attempted such projects (Fig. 1). They are: (1) the Simandou Project in the Republic of Guinea, (2) the Global Alumina Project (GAP) in the Republic of Guinea, (3) the Bumbuna Hydroelectric Project (BHP) in Sierra Leone, and (4) the Lom Pangar Dam in Cameroon. For each project, we researched all publicly available documents that included information on measuring and mitigating impacts on great apes and their habitat. Table 1 provides a list of those documents, available as of May 2014. We are also aware of other projects that have considered, or are considering, the use of offsets to compensate for residual damage to ape habitat in Africa. We focus on these four case studies because they are those of which the authors have the most direct experience. It will be important to eventually expand this type of analysis to include a larger set of projects. The current analysis, however, is an important first step. Table 2 provides a summary of predicted impacts on great apes and proposed mitigation measures for each project.

**Table 1:** Publicly available documentation for each site.

Project	Country	Document type	Source
Simandou Project	Guinea	Social and EnvironmentalBaseline Study 2010	http://ifcext.ifc.org/ifcext/spiwebsite1.nsf/0/A87B7EA570082C41852578E700569CED/$File/Vol.%20D_Biodiversity%20baseline_FINAL.pdf
		Social and EnvironmentalImpact AssessmentChapter12. Biodiversity	http://www.riotintosimandou.com/documents/Mine/M_Ch12_TerrBiodiv_EN.pdf
		Social and EnvironmentalImpactAssessment Annex12.E.West AfricanChimpanzee -SupplementaryBaseline andImpact Assessment Information	http://www.riotintosimandou.com/documents/Mine/M_An12E_Chimp_EN.pdf
		Environmentaland SocialAction Plan July 2013	http://ifcext.ifc.org/ifcext/spiwebsite1.nsf/0/A87B7EA570082C41852578E700569CED/$File/Simandou%20Project%20ESAP%20July%202013%20FINAL.pdf
Global AluminaProject	Guinea	Social andEnvironmentalAssessment	http://ifcext.ifc.org/IFCExt/spiwebsite1.nsf/78e3b305216fcdba85257a8b0075079d/8a0ee1048673cb16852576ba000e2cac?opendocument
		Critical HabitatAssessment2008	http://ifcext.ifc.org/ifcext/spiwebsite1.nsf/0/8A0EE1048673CB16852576BA000E2CAC/$File/Guinea%20Critical%20Habitat%20Assessment%20Report.pdf
		Action Plan 2008	http://ifcext.ifc.org/ifcext/spiwebsite1.nsf/0/8A0EE1048673CB16852576BA000E2CAC/$File/Post%20Comm%20Action%20Plan%20FINAL%20FINAL290808.pdf
Bumbuna	SierraLeone	EnvironmentalImpactAssessment 2005	http://www-wds.worldbank.org/external/default/WDSContentServer/WDSP/IB/2005/03/10/000012009_20050310135611/Rendered/PDF/E10930V.02.pdf
		2005	http://bumbuna.sl/admin/images/news/ESAP%20M4%20Draft%20Final%20Report%20for%20transmission%20JJ%2005.03.09.pdf
		Environmentaland SocialAdvisory Panel Report 2010	http://www.bumbuna.sl/admin/images/news/ESAP%20M1%20-%20Final%20Report%2010.11.04.pdf
		ESAP missionreport 2010	http://www.bumbuna.sl/admin/images/news/ESAP%20M5%20Draft%20Final%20Report%20ver%202%20JJ%2003%2011%2010.pdf
Lom Pangar	Cameroon	Social and EnvironmentalImpactAssessment 2010	http://www.edc-cameroon.org/IMG/pdf/sde/ANNEXE%204%20PNDD%20projet%20110111.pdf
		Environmentaland SocialAssessments 2011	http://www-wds.worldbank.org/external/default/WDSContentServer/WDSP/IB/2012/03/07/000350881_20120307112131/Rendered/PDF/673550BR0P11400ffiicial0Use0Only090.pdf

**Table 2:** Summary of predicted project impacts on great apes and proposed mitigation measures.

Project	Country	Type of project	Main Projectcomponents	Estimated # ofapes possiblyaffected byproject activities	Main predictedimpactson apes	Main proposedmitigationmeasures for apeconservation	Proposedoffsetsite
Simandou	Guinea	Iron-oremining	(i) An open-pit iron-ore minein the Simandou mountainrange; (ii) approximately 670 kmof railway across Guinea totransport ore to the coast;(iii) a new port facility;(iv) associated infrastructure, incl.housing, roads, quarries, powergeneration and distribution	<50 at the mine site andunknownnumber along therailway	Open-pit mining will remove anarea approximately 6–8 kmlong, 1–1.5 km wide and300 m deep. One quarter of thecore of the chimpanzees’ rangewould be permanently andirrecoverably lost to mining	(i) Improving control ofhunting; (ii) protectinghabitat within the chimpanzee’scurrent range; iii) creatingadditional habitat forchimpanzees both prior toand during mining activities	Undecided
Global AluminaCorporation	Guinea	Bauxitemining	(i) Mine sites in the 690-km^2^concession in northwest Guinea;(ii) an alumina refinery;(iii) a steam and power plant;(iv) a port facility 82 km fromthe refinery; (v) additionalinfrastructure on the concession	50–>100 in247.6 km^2^ of2008 CriticalHabitat surveyarea	Information not available	Information not available	Undecided
Bumbuna	SierraLeone	Hydroelectricdam	(i) An 88-m high asphaltconcrete-faced rock-fill dam;(ii) a water intake structure;(iii) two spillways with associatedtunnels; (iv) an above-groundpowerhouse with two 25-MWturbo-generator units; (v) a 30-kmwide, Y-shaped reservoir	33–58	(i) loss of natural resources forthe estimated four communitiesof about 33–58 chimpanzeesusing the area to be flooded;(ii) an increase in human-wildlifeconflict as farmers and wildlifeare forced closer together byreduction in available land;(iii) the prevention of movementof chimpanzees across the SeliRiver and between chimpanzeecommunities on each side of theriver resulting in a decrease intheir genetic viability	(i) Initiation of monitoringand awareness programsthroughout the catchmentarea; (ii) incorporation ofspecific conservation activitiesinto a Watershed Management Plan,including hunting controls,environmental awareness,fire control and zonation togive more protection to themost important remainingforest patches; (iii) creationof a Wildlife ConservationArea within the catchment	Loma MountainsNational Park
Lom Pangar	Cameroon	Hydroelectricdam	(i) A 50-m high dam located onthe River Lom in Cameroon’sEast Region; (ii) a 610-km^2^reservoir area; (iii) a hydroelectricpower plant; (iv) a transmissionline; (v) a rural electrificationscheme along this transmissionline.	About 990 gorillas are locatedin the greater Deng Deng area,with over 50% of thispopulation resident in DengDeng NP itself; the others arelocated in the ForestManagement Unit UFA 10–65and in the Belabo Forest.	The key impacts identified by theproject include: (i) loss of naturalhabitat due to flooding andinfrastructure footprint; (ii) riskof reducing the viability of adistinct population of gorillasand other Red-Listed species;(iii) the risk that constructionactivities will induce significantloss of natural habitat;, and(iv) the risk of future habitat lossdue to increased human pressuresin the region.	Minimization: an adjustmentto the pipeline route toavoid central Deng Dengand other dense forestareas and an offset site.Compensation: i) Extensionof Deng Deng National Parkby 9,000 ha, and creation ofprotection corridors to allowmovement of gorilla populationsin and out of Deng DengNational Park;ii) Conservation managementprograms in two ForestManagement Units;iii) Creation of communityforests (e.g. Belabo Forest) tosupport sustainable livelihoodsand gorilla protection;iv) Development of mechanismsto control access to the region,especially during constructionphase to prevent illegal logging;v) development of a financingmechanism to supportconservation managementand maintenance ofecoguards.	Forest areas aroundDeng Deng NationalPark perimeter

### The Simandou Project, Republic of Guinea

Simfer is a Guinean-registered company and holder of an iron-ore mining concession called the “Simandou Project” in the Simandou mountains of Southeast Guinea. The “Simandou Project” partners include the Republic of Guinea, Rio Tinto, Aluminium Corporation of China (“Chinalco”), and the International Finance Corporation (“IFC”). The proposed Simandou Project includes: (i) an open-pit iron-ore mine in the Simandou mountain range; (ii) approximately 670 km of railway across Guinea to transport ore to the coast; (iii) a new port facility; and (iv) associated infrastructure, such as housing, roads, quarries, and power generation and distribution.

The Simandou Project has been collecting data on chimpanzees in the Pic de Fon Classified Forest since 2007 to guide the development and implementation of a mitigation plan–the Pic de Fon Management Plan and the Simandou Project Biodiversity Offsets Strategy. The study has identified an estimated 36–46 western chimpanzees (*Pan troglodytes verus*) living in the forest in the Simandou range dispersed across one or two communities. The number of chimpanzees along the railway is unknown, but 2,750 chimpanzee nests were recorded along the rail study area. The Social and Environmental Impact Assessment (SEIA) outlines the range of impacts on chimpanzees caused by mining activities and forecasts “best-case” and “worst-case” scenarios, dependent on the number of communities of chimpanzees and the relationships between them. Assuming that there are two separate communities, the worst-case scenario predicts a high degree of chimpanzee mortality when the communities are forced together as they lose habitat and move away from mining activities. An estimated 25% of the core of the chimpanzees’ range would be permanently and irrecoverably lost to mining.

Mitigation proposed for the chimpanzees in the Simandou mountains includes controlling hunting, protecting habitat currently within the chimpanzee’s range that will not be lost to mining, and creating additional habitat for chimpanzees both prior to and during mining activities. The SEIA predicts that, despite mitigation efforts, the sub-montane forest habitat where chimpanzees are living will be impacted, and the project is therefore investigating an offset site to compensate for residual damage to this unique habitat and other species living there. Simfer has formed a technical group called the Simandou Offsets Working Group with representatives from Simfer, the Environment Ministry, and the NGO Guinée Ecologie.

On 26^th^ May 2014, the Government of Guinea, Rio Tinto, Chinalco and the IFC, signed an Investment Framework for Blocks 3 and 4 of Simandou, which now makes it the largest combined iron-ore and infrastructure project ever developed in Africa.

### Global Alumina Project (GAP), Republic of Guinea

The “Guinea Alumina Project” (GAP) is a 690-km^2^ bauxite-mining concession in northwest Guinea that was developed in 2008 by the Global Alumina Corporation–a joint venture of BHP Billiton, Global Alumina, Dubai Aluminum Company Ltd., and the Mubadala Development Company. The initial exploitation zone was approximately 100 km^2^, to be operational for 16 years. GAP includes a mine, an alumina refinery, a steam and power plant, a port facility, additional infrastructure on the concession, and a port facility 82 km from the refinery. GAP expected to employ about 12,000 workers during the 4-year construction period and more than 2,100 employees thereafter.

GAP originally hired the company Bechtel to conduct a chimpanzee Critical Habitat study, but the IFC requested that the study be redone. A second Critical Habitat study was conducted, although only two weeks were allowed for the fieldwork. The second study estimated that a minimum of 50 chimpanzees was living in the area surveyed [48]. GAP then hired the Wild Chimpanzee Foundation (WCF) to conduct a longer-term survey of chimpanzees covering the entire concession, to assist with the design and implementation of a conservation management plan, and to look for conservation sites to offset the expected decrease in the chimpanzee population caused by the project. Information on the predicted habitat and population decline as a result of project activities and on suggested mitigation for mining activities on chimpanzees is not publicly available.

The Global Alumina Corporation (GAC) is now a branch of the newly formed Emirates Global Aluminum (EGA) founded by Mubadala and DUBAL, and predicted to become the fifth-largest aluminum company in the world by production in 2014 [49].

### The Bumbuna Hydroelectric Project (BHP), Sierra Leone

The Bumbuna Hydroelectric Project (BHP) Phase I is a 50-MW, water regulation and hydropower facility located on the Seli River near Bumbuna, Sierra Leone. The project consists of an 88-m high asphalt concrete-faced rock-fill dam, a water intake structure, two spillways with associated tunnels, an above-ground powerhouse with two 25-MW turbo-generator units and a 30-km wide, Y-shaped reservoir. The USD 91.8 million project was funded by the African Development Bank, the Government of Italy, Organization of the Petroleum Exporting Countries (OPEC), the Netherlands Clean Development Facility, the World Bank, UK DFID, and the Government of Sierra Leone.

An Environmental Impact Assessment (EIA) was completed in 2005 [50]. An Environmental Management and Mitigation Plan (EMP) was designed to include the construction phase, and the mitigation and monitoring activities associated with the first component, and includes the preparation and initiation of the Bumbuna Watershed Management Plan. In 2004, the World Bank assembled an Environmental and Social Advisory Panel (ESAP) to review these studies. In 2006, a full biodiversity assessment was conducted as a follow-up to the BHP EIA. As part of this, the size, distribution and socio-ecology of the chimpanzee population in the immediate catchment area was studied to determine the impact on chimpanzees of the filling of the reservoir and the associated loss of riparian forest [51]. The 2006 study estimated that four communities totaling 33–58 chimpanzees used the area to be flooded and that the main impacts would be (1) loss of the chimpanzees’ natural resources, (2) an increase in human-wildlife conflict as farmers and wildlife are forced closer together by reduction in available land, and (3) the prevention of movement of chimpanzees across the Seli River and between chimpanzee communities on each side of the river. The study predicted that these effects could result in reduced viability of the chimpanzee population in the BHP catchment over the long term due to genetic isolation [51]. Recommendations for the mitigation and offset of these impacts included initiating a monitoring and awareness program in the catchment area, and incorporating conservation activities such as hunting controls, environmental awareness, fire control and zonation to give more protection to the most important remaining forest patches in a Watershed Management Plan. The 2006 study also suggested the establishment and management of a Wildlife Conservation Area in the catchment area, to be called the Bumbuna Conservation Area (BCA), to preserve and protect biodiversity in the Bumbuna watershed and to serve as a biodiversity offset. The ESAP’s view was that this BCA would not be very effective for chimpanzees, given the small size of the area and the many people living and farming within it. It therefore recommended creating an offset conservation area outside the catchment area. It also recommended that the Loma Mountain Non-Hunting Forest Reserve, an even more diverse 396-km^2^ forest, 50 km from the dam with a population of approximately 1,065 chimpanzees [52], be upgraded to national park status as additional compensation.

The dam construction was completed and the reservoir area flooded in 2009. In 2012, Loma Mountains National Park (LMNP) was proclaimed, although parliamentary approval is still pending. Measures for the park have included: (a) posting and training staff; (b) providing equipment and materials; (c) conducting consultations on the park boundary, (d) resurveying and physically demarcating the boundary; (e) completing Reserve Settlement Courts Sittings; (f) completing a Process Framework, including a socio-economic baseline study; (g) developing a Management Plan; and (h) developing a provisional 5-year budget. These activities were funded by the original loan guarantee turned into a grant to support environmental and social mitigation at Bumbuna, as well as a GEF project that has been building government capacity to conserve national biodiversity. Sustainable financing for the LMNP has not yet been secured [53]. Since no long-term monitoring of chimpanzees was undertaken, the responses of the chimpanzees to the flooding remain unknown. A new Bumbuna Phase II project has now been launched that will expand the Bumbuna hydroelectric station and flood an even greater area.

### Lom Pangar Hydropower Project (LPHP), Cameroon

The Lom Pangar Hydropower Project (LPHP) consists of a regulating, 50-m high dam located on the River Lom in Cameroon’s East Region, a 610-km^2^ reservoir area, a hydroelectric power plant, a transmission line, and a rural electrification scheme along this transmission line. Estimated costs for the LPHP are USD 393 million from the African Development Bank (AfDB), the Central African States Development Bank (BDEAC), the European Investment Bank (EIB), the French Agency for Development (AFD), the Government of Cameroon, and the World Bank.

The Environmental and Social Assessment noted that the main impact would be the flooding of natural forest, that none of the flooded habitat was critical, but that the dam site was located next to portions of the Deng Deng forest that included critical habitats and populations of the Critically Endangered (CR) Western lowland gorilla (*Gorilla gorilla gorilla*) and the Endangered (EN) Central chimpanzee (*Pan troglodytes troglodytes*). About 990 gorillas are located in the greater Deng Deng area, with over 50% of this population resident in Deng Deng National Park itself; the others are located in the Forest Management Unit UFA 10–65 and in the Belabo Forest [54]. Suggested mitigation included an adjustment to the pipeline route to avoid the central Deng Deng and other dense forest areas and control of access to the area to prevent illegal logging during the construction phase. Nonetheless, the project was predicted to have significant impacts on natural habitats that should be compensated. Studies funded by the AFD and carried out by the Wildlife Conservation Society (WCS), estimated a population size of several hundred gorillas in the greater Deng Deng area, which included a communal forest and a logging concession. As a result, a decision was made that the company would provide a high-level compensation package designed to strengthen protection of the newly designated 580-km^2^ Deng Deng National Park, extend its boundary, and establish a corridor to other forest areas [55]. A third-party analysis indicated that an annual payment of USD 700,000 per year would be required to provide sufficient financing to meet the conservation management needs in the region. The activities in Lom Pangar are considered compensation rather than an offset since no efforts were made to quantify losses or gains to biodiversity and offset them to achieve no net loss.

## Analysis

In the following, we analyze to what extent these field projects follow international best-practice principles for biodiversity offsets and whether these principles adequately generate conservation benefits for great apes in light of their EN and CR status, shrinking habitats, and vulnerability to disturbance. Table 3 provides a summary of our findings.

**Table 3:** Summary of project’s implementation of international best practice principles for biodiversity offsets with respect to great ape conservation.

Project	Limitsto whatcan be offset	Adherenceto themitigation hierarchy	Additionalconservationoutcomes	Landscapecontext	No net loss	Long-termoutcomes
Simandou	Chimpanzeesare notconsidered beyond thelimit to what can beoffset.	All possible mitigationon site and changes tothe mining plan will theoretically be followedbefore offsets areconsidered for residual damage to chimpanzeesand their habitat.	Unknown.	The potentialoffset sitesbeing consideredare priorityareas for chimpanzees.	No net loss mayoccur in medium-term but there willprobably be ashort-term andpotentially long-termloss of chimpanzees.	Dependent on financingmechanisms,coordination with other offsetprojects and whethercommitmentto offset site is in perpetuity.Long-term outcomes thereforeunknown at this point.
Global AluminaCorporation	Chimpanzeesare notconsidered beyond thelimit to what can beoffset.	Some infrastructure and originalmining planalready inplace before2008 Critical Habitat study was conducted.	Unknown	Unknown	Unknownbecause offsetsite is stillnot certain.	Dependent on financing mechanisms,site selection, coordination withother projects. Long-term outcomesare therefore unknown at thispoint.
Bumbuna	Chimpanzeesare notconsidered beyond thelimit to what can beoffset.	Site selection andinfrastructure werealready in place before the ESAP was engaged.	Yes, if assumption ofbaseline decline ofchimpanzees in LomaMountains is correct.No, if assumption isincorrect. Yes, if Loma Mountains protected inperpetuity, but no if not.	The Loma MountainsNational Park is a priority area forchimpanzees.	Unknown. Specific calculations onlosses and gains of individualchimpanzees were not made.	No
Lom Pangar	Chimpanzeesand gorillasare not consideredbeyond the limit towhat can be offset.	No. Mitigation hierarchywas not specificallyapplied.	Yes, if assumption ofbaseline decline ofchimpanzees inDeng Deng is correct. No, if assumptionis incorrect. Yes, if DengDeng National Parkprotected inperpetuity, andthe adjacentareas are effectively managed, but no if not.	Deng Deng isa priority areafor gorillasand chimpanzeesand studiesindicated that anarea largerthan Deng Dengforest neededprotection andmanagementand thecompensationproject wasdesigned to address that.	Unknown. Specific calculations onlosses and gains of individualchimpanzees and gorillas were notmade. Key concern was to maintaingorilla population in the region.	Dependent on both companycompliance with financingcommitments (30 year annualpayments) and securing longer-term financing, and whetherDeng Deng will be protected inperpetuity.

### Limits to what can be offset

It is generally accepted that there are limits to what can be offset [36], [38], [39]. Some residual impacts on biodiversity cannot be fully compensated for by a biodiversity offset given the irreplaceability or vulnerability of the biodiversity affected [36]. Ecological criteria used to make decisions on where biodiversity offset limits should be drawn include: “levels of conservation concern”, “magnitude of the estimated residual impact”, and “opportunity and feasibility of offsets on the ground” [38], [39].

All great apes are listed as EN or CR [13]. They are highly vulnerable to habitat disturbances due to their life history, behavior and susceptibility to human diseases [9], [56]. They are important seed dispersers and play a role in shaping forest structure [57], [58]. Due to the high degree of their “impactability” and low degree of their “offsetability”, (i.e. their vulnerability and irreplaceability), these ecological considerations advocate in favor of an extremely high threshold for offsetting apes.

In addition to these ecological factors, offsetting great apes raises serious ethical questions. Great apes are our closest living relatives [59], [60], exhibit many of the same emotions as humans [61], [62], practice tool-use [63], hunt cooperatively [64], [65], and show evidence of culture and traditions [62], [66] as well as a capacity for language [68]. Some human communities in the region have religious, cultural and traditional taboos against hunting and eating great apes because of their close resemblance to humans [69]–[72]. We contend that from an ethical standpoint also, offsets for apes should require an extremely high threshold.

The IFC [44] recognizes both ecological and ethical values, and theoretically sets high standard thresholds for offsetting apes and ape habitat. IFC Guidance Note 6 divides Critical Habitat into two tiers with the likelihood of project investment in a Tier 1 habitat substantially lower than in a Tier 2 habitat. A footnote in Guidance Note 6 states that “special consideration should be given to great apes given their anthropological and evolutionary significance in addition to ethical considerations. Where populations of CR and EN great apes exist, a Tier 1 habitat designation is probable” [44].

In practice, however, the presence of apes in a project area does not seem to have deterred companies, governments or funders from investing in activities that will be detrimental to great ape habitat and likely to result in their decline. Both the GAP and the Simandou project are located in areas of Critical Habitat for chimpanzees, and both have received funding from the IFC. The BHP and the Lom Pangar Dam projects were financed by the World Bank, and both are considering or have implemented offsets or compensation projects for negative impacts to great ape habitat. In summary it seems that none of these projects have considered great apes to be beyond the limits of what can be offset.

### Adherence to the mitigation hierarchy

BBOP Principles [36] emphasize that biodiversity offsets are only appropriate after compliance with the mitigation hierarchy; that is, after avoidance, minimization and on-site rehabilitation measures have been exhausted [36]. Biodiversity offsets are therefore a mechanism of last resort [36], [43], [46]. In two of the projects profiled above, mitigation measures were not designed until they were already under way, resulting in the need for more off-site compensation than if mitigation measures had been included from the onset. For example, much of the BHP infrastructure was already completed when the ESAP was engaged, and the ESAP concluded that mitigation options for chimpanzees impacted by the dam were limited given that the dam site had already been selected [51]. Similarly in the case of the GAP, a Critical Habitat study for chimpanzees was not requested until the IFC was approached for a loan in 2008 [48], when much of the infrastructure already existed. The concept of “avoidance” was better integrated into the Simandou and Lom Pangar projects. For Simandou, the mining infrastructure development and plans for the sequence of exploitation activities were adapted to reduce impacts on chimpanzees. For Lom Pangar, costly mitigation measures were undertaken, including rerouting the pipeline to avoid the center of Deng Deng.

### Additional conservation outcomes

BBOP states that a biodiversity offset should achieve additional conservation outcomes beyond results that would have occurred had the biodiversity offset not taken place [36]. The most common form of “additionality” in countries with offset policies is habitat restoration [31]. Biodiversity offset best practices also state that biodiversity offsets can achieve “additionality” by protecting areas where there is imminent or projected loss of biodiversity [36], [73]. Two of the projects we examined based additionality on “averted loss” by updating the protected status of an area; the Loma Mountains National Park in Sierra Leone for BHP, and the Deng Deng National Park in Cameroon for the Lom Pangar Dam, and in the case of Lom Pangar extending areas for conservation around the park to protect great ape habitat. Both GAP and the Simandou project have provided a short list of potential offsets sites, indicating that their offsets will also consider “averted loss” as the counterbalance to actual loss on site. In these cases “no net loss” is working from an assumption of a pre-existing baseline rate of loss, assuming that habitat in the offset site is under threat or will be in the future [74]. This may be true given how many forests are threatened throughout the range of great apes. The result remains nonetheless a net loss of habitat against the extent and condition of that habitat at the time the project is implemented [73].

Maron *et al.* [31] emphasize that calculating the expected benefit of a conservation action–such as the purchase of a new reserve–requires “explicit estimation of the change in conservation value (e.g., population size of a threatened species) both with and without the action taking place, and calculation of the difference between these two scenarios. It is difficult to accurately (1) ascertain a baseline number of apes, (2) estimate the magnitude of change in ape numbers that would have occurred without project activities, (3) predict the magnitude of a population decline resulting from project activities; and (4) determine how much compensation is appropriate based on (1), (2) and (3). It is easier to estimate numbers of apes than of some other species given that their conspicuous nests can be used as indices of abundance. However, it is still extremely difficult to estimate numbers accurately [75]. Few studies have assessed the long-term impacts of extractive industries and other forms of habitat disturbance on apes [14]. As a result, it is very difficult for projects to measure losses, gains and additionality. Nevertheless, such estimations are necessary [36], and there should be consistency as to how they are generated and at what scale they are being assessed.

The projects we examined approached the challenge of measuring “additionality” in different ways, without common standards for measuring losses or gains. For the GAP, researchers from WCF proposed a mathematical formula to predict losses of chimpanzees, and gave a dollar value that companies should pay to compensate for this loss [76]–[78]. For BHP, it was predicted that all 33–58 chimpanzees would be impacted, but it was not specified whether “impact” would result in their death. The Loma Mountains compensation was assumed to be far greater than the loss [51]. In the case of the Lom Pangar project, the project appraisal indicated that the project would have significant and irreversible environmental impacts, including the loss of natural habitat and the risk of reducing the viability of a distinct population of gorillas and other Red-Listed species. Bolstering the protection of the national park and designating new areas for conservation management were considered additional.

Another challenge in estimating losses, gains and additionality under the current framework is that methodologies do not take into account the cumulative impacts of multiple projects, which can be far greater than the sum of the impact of individual projects. This lack of information on cumulative impacts is in part a result of offsets being funded, designed and implemented on a project-by-project basis.

Since great apes are distributed across equatorial Africa, they are likely present in or around many mining concessions. When concessions are adjacent to each other, there will be few available locations for apes to escape the mining activities. For example, the GAP concession is adjoined by a concession held by Compagnie des Bauxites de Guinée (CBG) to the east and a concession held by Russian Aluminium (RUSAL) to the north. Chimpanzees fleeing noise and other human disturbance in the GAP concession may not have accessible undisturbed habitat to move into. These projects state that they have conducted cumulative impact assessments, but these generally refer only to the direct effects on the environment from their own activities and not their impacts in combination with the activities of other companies. Tools exist to aid such an analysis. They include the Cumulative Impact Assessment (CIA), the Regional Cumulative Impact Assessment (RCIA), and the Strategic Environmental Assessment (SEA). The IFC recognizes that the “CIA should be an integral component of a good environmental and social impact assessment (ESIA) or a separate stand-alone process”. They also recognize, however, that the “CIA is evolving and there is no single accepted state of global practice”. In addition, the IFC Performance Standard 1 “does not expressly require, or put the sole onus on, private sector clients to undertake a CIA” [79]. Without better coordination and accounting for cumulative impacts, the risk is that offsets and compensation projects will be insufficient to offset the total cumulative loss of EN and CR species nationally or regionally over time, leading to overall species loss.

### Landscape context

Several authors have encouraged biodiversity offsets to be designed in a landscape context [38], [39]; however, there is little guidance regarding how this may be accomplished. The Simandou Project SEIA explicitly stated that the project would ensure that offsets were aligned with national biodiversity priorities. Both the Simandou Project and the GAP are considering sites identified in an IUCN action plan for West African chimpanzees [80]. Studies conducted in the Loma Mountains and Deng Deng forests determined both these areas to be important for apes and biodiversity in general. Thus these biodiversity offset and compensation sites appear to be located in national priority sites for these species and seem to be complying with this best practice principle. However, when offset projects are designed on a project-by-project basis without coordination or integration with other offset or compensation projects or other conservation initiatives, opportunities for aggregating sites are missed. Aggregating protection of larger areas of habitat, or connected forest patches, would have a better chance of maintaining viable populations of apes over the long term. Operating on a project-by-project basis does not rule out placing the offset location into a larger landscape context but could still result in the protection of multiple small, isolated and vulnerable sites, impacting the ‘additionality’ potential of the offset project.

### No net loss

One of the reasons that offset design and implementation continue to be *ad hoc* is that there are differing interpretations even as to the meaning of “no net loss” [37], [38]. The IFC defines no net loss as: “the point at which project-related impacts on biodiversity are balanced by measures taken to avoid and minimize the project’s impacts, to undertake on-site restoration and finally to offset significant residual impacts, if any, on an appropriate geographic scale (e.g., local, landscape-level, national, regional)”. As discussed above, the challenges of accurately estimating the losses and gains of individual apes are enormous. Even if this were possible, and even if a project could result in an increase in ape numbers at a particular site, a small local increase would not necessarily contribute to the viability of the population as a whole. The worst-case scenario would be that the criterion of no net loss would merely result in many isolated offset projects protecting isolated individuals, groups or communities. While that may counterbalance losses of individual apes from the activities of individual projects, it does not necessarily contribute to protecting viable populations that would survive in the long term. This is again due to offsets being designed and implemented on a project-by-project basis.

By not coordinating with other projects, the projects we examined may have missed opportunities to aggregate offsets and create larger, more robust offset areas. Species viability in forest patches depends on many factors, including the size and shape of habitat patches and connectivity between patches. Not only does fragmentation disrupt the distribution of the species, it also affects the ecological processes that are part of the ecosystem [81]. Designing biodiversity offsets on a project-by-project basis could indeed temporarily result in a “no net loss” or “net gain”, but in the long term, species viability could be eroded if offset sites exist in isolation from each other.

### Long-term outcomes

BBOP addresses the need for long-term protection by emphasizing in their best practice principles that the outcomes of a biodiversity offset should last at least as long as the project’s impacts, and preferably in perpetuity [36]. The ability of a biodiversity-offset project to deliver long-term outcomes depends on both biological and financial factors. Above, we have already discussed the biological factors affecting long-term outcomes. When we examined the financial sustainability of these projects, we found no consistency in the way offsets and compensation projects for apes are being funded in Sub-Saharan Africa.

In the BHP and Lom Pangar projects, the intended source of conservation funding is revenue from electricity production, which would be disbursed to specific conservation projects on an annual basis. However, disruption of either dam’s operations would threaten this funding. In the case of BHP, problems with tariffs and the distribution system have curtailed profits and caused operational disruptions. In the case of Lom Pangar, the annual conservation payments were designed to last 30 years, but there are no specific payment guarantees to ensure the consistency of payments or any identified system for recourse if the project does not comply. This lack of financial security places compensation projects at risk, as well as the great ape populations whose security is dependent on effective conservation management. No information is currently available on how the GAP or Simandou offsets will be funded.

## Discussion

### Adherence to Biodiversity Offset Principles

Our review of these four projects that are investigating or already employing great ape offset or compensation projects demonstrates that the degree to which international best practices are applied is mixed. Despite the vulnerability of apes to disturbances, the long time it takes ape populations to recover from disturbances, their EN and CR status, and the ethical questions surrounding offsetting apes, all four projects assumed that great apes and their habitats could be at least compensated if not totally offset to achieve a no net loss. Following the mitigation hierarchy in most circumstances appears to have been challenging because project sites had already been selected using non-biodiversity criteria, and in some cases infrastructure development had already taken place, decreasing the options for mitigation and thus increasing residual impacts. Predicting losses, gains and “additionality” for apes is challenging, and most projects avoided making this calculation altogether. In all cases additionality was based on “averted losses”. The projects might achieve “no net loss” or even support small local increases in numbers (“net gain”) in isolated locations, but long-term outcomes of the two projects that have already been implemented are questionable given the uncertainty of the financing mechanisms and the fact that all offset or compensation projects were being designed on a case-by-case basis. Offset and compensation sites might be placed in priority locations, but without an overall offset strategy, opportunities are being missed for aggregating offsets in time and location and integrating them with species conservation objectives and other national biodiversity priorities. In summary, even though the projects we examined may result in temporary no net loss or even a net gain in species numbers, the current trajectory for great ape biodiversity offset projects is unlikely to result in no net loss over the long term. This indicates that, even if these projects adhere closely to international biodiversity offset principles, this will not ultimately generate a meaningful conservation outcome, and will not be sufficient to protect great ape populations over the long term, or contribute to species recovery.

Our assessment revealed three challenges that limit the effectiveness of efforts to compensate for impacts on great apes even if adherence to biodiversity offsets principles is improved. The first is that current great ape offset and compensation projects fail to account for cumulative development impacts at larger scales. The second is that great ape offset projects are less likely to make a meaningful contribution to great ape conservation if conducted on a project-by-project basis that results in multiple, isolated projects. This could occur even if offsets are placed in a larger, landscape context, since each location within the larger area could be disconnected from the other. The third is that biodiversity offset principles do not require offset projects to be fully operational and delivering the required biodiversity compensation before impacts from the development project occur. We suggest that a fundamental shift in the way offsets are designed and implemented is needed.

### National Offset Strategies as an alternative trajectory

The most effective means of ensuring that biodiversity offset projects adhere to existing international best practice principles and contribute more effectively to great ape conservation is to develop National Offset Strategies, supported by conservation trust funds with non-wasting endowments. This new trajectory would have the following advantages.

National Offset Strategies would provide a framework for managing biodiversity offsets in a coordinated and transparent manner, consistent with national biodiversity strategies, including national protected area system plans and species recovery plans. Coordinating offsets and compensation projects through a National Offset Strategy would help ensure that sites are selected strategically, providing synergies with other conservation areas and between offset sites, and ensuring contribution to a landscape-level approach to great ape conservation. It would also help to ensure compliance with the mitigation hierarchy at the outset and that offset sites are prioritized in terms of timing of investment and location.

National Offset Strategies would maximize conservation benefits, for example, by establishing connectivity, buffering existing conservation areas, creating larger areas by aggregating offsets, or by selecting sites in different parts of the country, especially where several distinct and unconnected areas may be needed to buffer against the spread of disease such as Ebola–a major threat to humans and great apes in Africa [82].

In addition to increasing the conservation benefits, National Offset Strategies would also benefit project developers in a number of ways. They could establish a common set of rules, leveling the playing field, helping protect companies from reputational risk, and raising standards of industrial development projects. They would not limit offsets to those companies applying for funding from financial institutions with offset standards or companies with internal offset standards. National Offset Strategies would also allow companies to entrust offset management and implementation to a permanent entity, such as a conservation trust fund, with the responsibility of funding and implementing the strategy, rather than assuming the burden and liability of managing offsets in perpetuity (conservation banks in the U.S. provide a similar function). Investing in aggregated offset sites identified by National Offset Strategies could also decrease transaction costs by pooling resources for formulating offset methodologies, biological surveys, priority setting, or conservation trust fund development, which would otherwise be incurred by developers. With coordinated planning, companies could also share infrastructure or take advantage of efficiencies that lead to greater avoidance and minimization of impacts before offsets are even considered.

National Offset strategies should be developed as a result of a science-based, multi-stakeholder process and should include the following components:

#### A species recovery goal for EN and CR species rather than no net loss

Given the worsening global species extinction crisis [83]–[85], the international goal of biodiversity offsets should not just be “no net loss” but rather to make a measurable contribution towards *recovery* of EN- or CR-listed species. The idea that biodiversity offsets should contribute to species recovery has precedent in the U.S. where biodiversity offsets originated. A key aspect of offset policy in the U.S. is that the Endangered Species Act (ESA) seeks to ensure that a species listed under the ESA “recovers”, that its conservation status improves to the point where it is no longer endangered (i.e. “in danger of extinction throughout all or a significant portion of its range”) or “threatened” (i.e. “likely to become an endangered species within the foreseeable future throughout all or a significant portion of its range”). Thus, the United States Fish and Wildlife Service (USFWS) develops “recovery plans” for endangered species listed under the ESA to guide conservation decisions for that species. A federal permit allowing an impact on endangered species is only granted if the development activity will not “appreciably reduce” prospects for both the survival and recovery of the species [86].

#### Articulation of and adherence to no-go zones

Some biodiversity values are not offsetable, either because of their vulnerability and irreplaceability or because of their location. Industrial activities, for example, should not be permitted in World Heritage Sites. National Offset Strategies could play a key role in helping to determine which locations and which species should be off limits because their biodiversity value cannot be offset.

#### Cumulative impact assessments Sectoral EIAs

The need for projects to take into account the cumulative impacts of neighboring projects impacting EN and CR species when designing offsets is a growing concern [25], [38], [87], [88]. We suggest that a nationwide assessment of the collective and cumulative impacts of planned and ongoing projects impacting EN or CR species should be part of the national offset design process, We also believe that assessment of cumulative impacts *before* offsets are designed should be an additional international principle as this could affect the magnitude of the offset required.

#### An opportunity for aggregating offsets

The challenge is to develop mechanisms to help ensure that biodiversity offset and compensation projects are implemented in the context of larger frameworks for endangered species conservation rather than on a project-by-project basis. “Conservation banking” was pioneered in the U.S. in part to address the problem of isolated biodiversity offset projects [89]. Conservation banks are lands that are conserved and permanently managed for threatened species. In exchange for permanently protecting and managing the land for the species of concern, the U.S. Fish and Wildlife Service (USFWS) agrees on a set number of habitat or species credits that bank owners may sell for that area. Developers whose projects have unavoidable adverse impacts on that species may purchase the credits from conservation bank owners to offset these impacts [90]. Conservation banks are expected to support a viable population of a species or contribute to the maintenance of a population by expanding an area managed for the species [89]. They provide an aggregated approach to offsets rather than a series of smaller, less viable projects. Conservation banks are permitted by USFWS, the same agency that also develops species recovery plans. Similar opportunities for aggregating offsets should be made available in National Offset Strategies.

#### Offset implementation to be complete before development occurs

The timing of biodiversity offsets can be an important factor in determining their success [38], [73], [91]. If development takes place before the offset is implemented, biodiversity may be lost [38], including loss of resources that are key to an EN or CR species’ survival (e.g., a tree that must reach maturity to bear fruit necessary for ape survival). Allowing development to proceed before the offset is complete also creates a significant risk to biodiversity if the offset project fails. To increase the likelihood of project success, it is therefore important that offsets are in place before development occurs. Bekessy *et al.* [73] argue for a biodiversity “savings bank” approach rather than a “lending bank” in which the public “owns all the risk of failure”, suggesting that “the biodiversity value of offsets should be realized before assets are liquidated”, and that assets can only be traded once it has been demonstrated that they have matured (reached ecological equivalence with whatever losses they are being traded against) ([73], p.153).

There is precedence for this approach in the U.S., where conservation banks must demonstrate measurable conservation benefits before issuing credits. The conservation benefit is then far less speculative than if offset activities are concurrent with development, an approach that experts in the U.S. have found usually results in biodiversity losses [88], [89], [92].

Because restoration of tropical rainforests is a very lengthy process [31], and because great ape population increases would not be apparent for many years given their slow reproductive rates, some may argue that demonstrating an increase in great ape numbers before development can occur is not realistic. At a minimum, however, we suggest that areas proposed for higher protected area status, should at least have been created and already have appropriate levels of trained staff, necessary equipment and secure long-term funding before any development is allowed to occur.

#### Long-term funding

Industrial projects impacting EN and CR species should also ensure guaranteed, permanent funding for offset projects specifically targeting EN and CR species. We propose that Conservation Trust Funds (CTFs) with non-wasting, or permanent, endowments (which already exist in many countries and could accommodate multiple offset projects) could be the most effective mechanism. CTFs provide independent and permanent entities that can assume responsibility and liability for financing, managing and evaluating offsets and compensation projects in perpetuity. This is an important function, as conservation project management is not part of the core business of most companies. CTFs for countries where trust creation is not possible legally, or where governance is weak, can be located offshore, while maintaining in-country management and operations to ensure multi-stakeholder representation and promote civil society.

#### Offset insurance sites

Due to the high risk involved in biodiversity offsets, particularly in countries with weak conservation capacity and governance concerns, but also as a result of natural disasters or other unforeseen causes for project failure [93], we propose that National Offset Strategies also identify “insurance” sites. Developers would then be required to invest in both offset and “insurance” sites in case an offset fails.

## Conclusion

Developing and enforcing a National Offset Strategy would help promote adherence to current best practice principles for biodiversity offsets and would be more likely than the current trajectory to contribute to species recovery objectives. Because offsets for many large-scale development projects need to be permanent, and because mechanisms for ensuring permanent protection on private lands may be lacking, the default is likely to be the creation of new protected areas, or the expansion or improvement of the management of existing protected areas. National Offset Strategies would, therefore, be likely to contribute to the establishment of well-managed national protected area systems containing a representative cross section of a country’s biodiversity.

Globally, the current rate of species extinctions is 1,000 times the predicted background rate of extinction [83]. Habitat degradation and conversion remain a leading cause of biodiversity decline [21]. A new approach to their design and implementation is needed if offsets are to be a useful tool for great ape recovery.

Here we have presented a new framework for designing and implementing biodiversity offsets that we believe has a greater likelihood of protecting EN and CR species in the long term. We stress, however, that caution should be exercised in the use of offsets as a tool for ape conservation until further field-based evidence of their effectiveness is available. Biodiversity offsets remain an unproven mechanism, and the risk of failure is magnified in countries with poor governance and recent histories of civil conflict. More time is needed to gauge whether offsets are truly contributing to no-net-loss objectives and to progress towards more “evidence based” approaches to offset design [94], even in developing countries where biodiversity offsets have been in use for some time [37], [72], [88], [91], [95], [96]. The international conservation community, development organizations, the private sector, and international lending banks should ensure not only a precautionary approach to the use of conservation offsets, but also that offsets are designed and implemented in the context of National Offset Strategies.
